# Supplementary material for: Evolution of Cu-In Catalyst Nanoparticles under Hydrogen Plasma Treatment and Silicon Nanowire Growth Conditions
Source: Nanomaterials (Basel). 2023 Jul 12;13(14):2061. doi: 10.3390/nano13142061 (PMC10384329; doi:10.3390/nano13142061)
Supplement: Supplementary file 1 [file nanomaterials-13-02061-s001.zip › nanomaterials-2454507-supplementary.pdf]

Evolution of Cu-In Catalyst Nanoparticles under Hydrogen Plasma Treatment and Silicon Nanowire Growth Conditions

Weixi Wang [1], Éric Ngo [1], Pavel Bulkin [1], Zhengyu Zhang [2], Martin Foldyna [1], Pere Roca i Cabarrocas [1], Erik V. Johnson [1] and Jean-Luc Maurice [1,*]

[1] Laboratoire de Physique des Interfaces et Couches Minces, École Polytechnique, CNRS, IPParis, 91120 Palaiseau, France; martin.foldyna@polytechnique.edu (M.F.)
[2] Laboratoire LuMIn, École Normale Supérieure Paris-Saclay, CentraleSupélec, Université Paris-Saclay, CNRS, 91190 Gif-sur-Yvette, France
* Correspondence: jean-luc.maurice@polytechnique.edu

Supplementary Information

Figure S1 shows a schematic diagram of a SiNW growth experiment in a PECVD reactor. The NW growth process is applied after the vacuum reaches 5×10^{-5} mbar in the PECVD chamber. The temperature of the substrate holder is firstly increased to 200 ° C in vacuum with a heating rate about 10 ° C/min for a hydrogen plasma treatment. The substrate holder usually overheats compared with the target temperature; therefore, we introduce the gas when the substrate holder is overheating and wait until the substrate temperature stabilizes at 200 ° C, then we ignite the hydrogen plasma for 2 minutes. Then we increase the temperature of the substrate holder in vacuum with the plasma off. The hydrogen + silane plasma is started when the temperature stabilizes at about 420 ° C for a SiNW growth process for 3 minutes. Then the chamber is cooled down with a hydrogen flow rate of 100 sccm. When the substrate temperature is below 80 ° C, we open the PECVD chamber to take out the sample for TEM characterization.

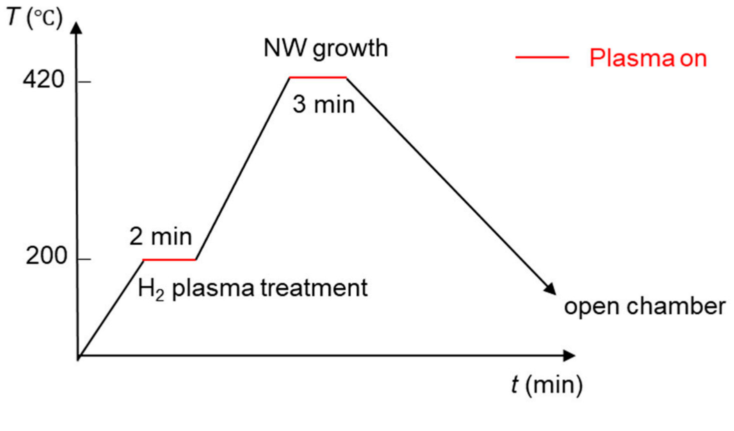

Figure S1. Schematic diagram of a SiNW growth experiment using 1 nm In/1 nm Cu catalyst in a PECVD reactor.

Figures S2 and S3 emphasize the influence of the plasma. In Figure S2, one analyzes the effect of plasma during Step II at 420 °C, taking the 200 °C H_2-plasma treatment as a reference (Figure S2a and d, S2a is the same as Figure 1b of the text). One compares a treatment at 420 °C for 3 min in vacuum (Figure S2b and e) with a H_2-plasma treatment at the same temperature and during the same time

(Figure S2c and f). The figure includes selected area diffraction patterns (SADPs) recorded in each case over an area of the order of the μm^2, delivering characteristic powder patterns (Figure S2d-f).

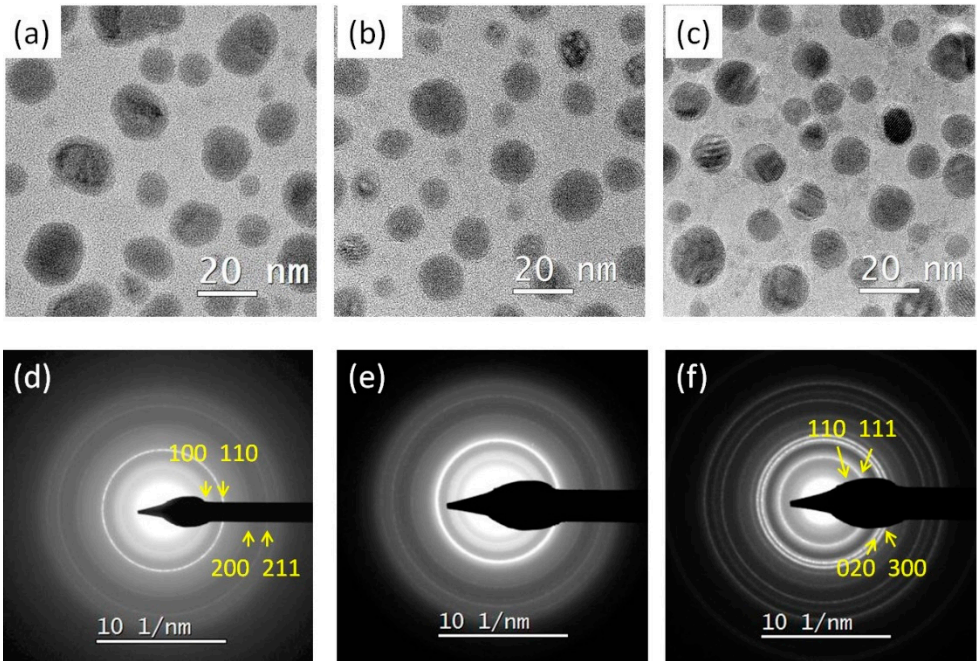

Figure S2. TEM of bimetallic catalyst NPs obtained with a nominal thickness of 1 nm In/1 nm Cu: (a,d) after H_2-plasma treatment at 200 °C; (b,e) after the same treatment followed by a 420 °C anneal in vacuum; (c,f) after a H_2-plasma treatment at 420 °C. (a-c) TEM images; (d-e) corresponding SADPs recorded over large areas. The pattern in (d) is indexed in terms of the Cu_4In-β phase (see text). Note the similarity between (d) and (e), signaling the absence of phase change during the annealing at 420 °C without plasma, and the difference of (f) with respect to the two others: with new rings, which are indexed in terms of $Cu_{11}In_9$, and a significantly lower background, signaling a decrease of amorphous structures.

In the reference 200°C H_2-plasma-treated sample, this powder pattern exhibits characteristic features of the cubic-centered Cu_4In β phase: d_{100}=0.301 nm; d_{110}=0.213 nm; d_{200}=0.151 nm; d_{211}=0.123 nm [1] (ICSD file #109480). Quite remarkably, this phase is normally stable above 574°C [2]. Let us note that these rings could also have been interpreted in terms of Cu_2O or the Cu_2In δ-phase, both stable at room temperature. However, the former is unlikely after a H_2-plasma treatment, and the latter would also deliver a reflection at 0.26 nm, with an intensity higher than that of the 0.30 here, which is not detected. Thus, this normally metastable phase at 200 °C would be stabilized by the H_2-plasma and the liquid In shell. Interestingly, annealing this 200 °C plasma-treated sample at 420 °C in vacuum does not modifies its structure, which keeps the metastable Cu_4In phase (Figure S2e). In contrast, treating a same deposit with the same H_2-plasma, but directly at 420°C, delivers the $Cu_{11}In_9$ phase [Figure S2f, (ICSD file #238715)], which also differs from the Cu_7In_3-δ found when SiH_4 is present in the plasma. Let us note, in this 420 °C H_2-plasma treated sample, the large decrease of the background signal present in both the sample treated at the same temperature without plasma (Figure S2e) and in that only treated at 200 °C (Figure S2d). This decrease is most likely related with the diffusion of a significant part of the amorphous indium, present in the latter case (Figure 1c in the paper), into the new $Cu_{11}In_9$ compound.

In Figure S3, one compares the 2-min H_2-plasma-treated nanoparticles (NPs) (Fig. S3c; which is fig 1b in the text and fig. S2a above) with a deposit that has undergone only the 2-min 200 °C treatment without plasma (Fig. S3b), and with the as-grown state (Figure S3a; Fig 1a of the text).

At 200 °C without plasma (Figure S3b), the NPs have started to coalesce but much less than with plasma (Figure S3c), and their worm-like shape has remained similar to that in the as-grown state (Figure S3a).

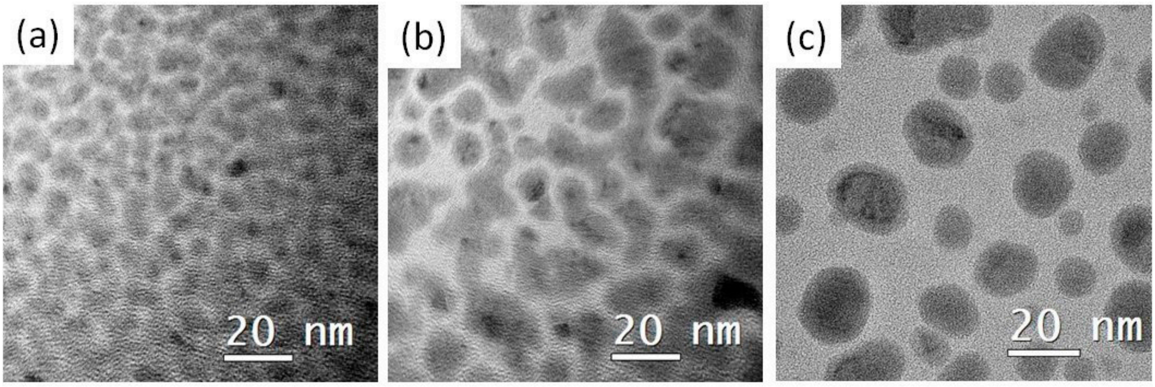

Figure S3. TEM images of the bimetallic catalyst NPs obtained with a nominal thickness of 1 nm In/1 nm Cu: (a) as deposited (Fig. 1a of the text); (b) annealed at 200 °C for 2 minutes without hydrogen plasma; the structure keeps including significant amounts of amorphous matter (most of lighter areas); (c) same treatment but with H_2-plasma (Fig. 1b of the text).

Energy-dispersive X-ray spectroscopy (EDX) was carried out on several samples. We tried, for instance, to record maps of the two elements in the catalysts after different treatments: unfortunately, the beam damage was such that, most of the times, the NPs were lost after a few scans, making it impossible to obtain such data. After treatments at 420 °C, however, some records could be made, presented in Figure S4. Figure S4a,b shows EDX maps of nanoparticles after Step I (H_2-plasma at 200°C) + 420°C vacuum anneal, where a core-shell structure exists before mapping. Note that the elements have been mixed up by the beam, leaving only a narrow In-segregation at the surface (Figure S4b). This mixing is consistent with the metastability of the Cu_4In phase present in this particle before the analysis (cf. Figure S3b,e). Figure S4c,d presents a static analysis carried out on a catalyst particle staying at the top of a SiNW. A quantitative treatment of the spectrum in Figure S4d gives

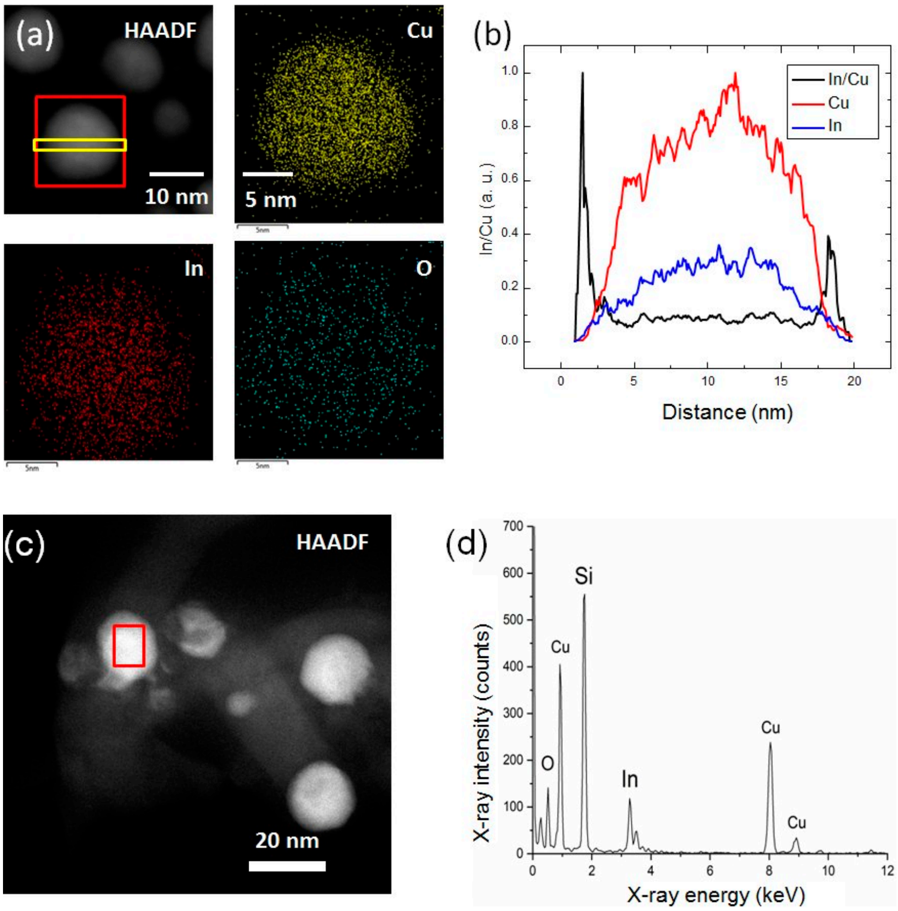

Figure S4. (a,b) EDX mapping of a NP in the 1-nm In/1-nm Cu deposit treated with the 200°C plasma followed by the 420°C vacuum annealing; (a) the EDX maps were recorded over the red square in the high-angle annular dark-field (HAADF) image; (b) normalized atomic amounts of Cu (in red) and In (in blue), over the profile indicated by the yellow rectangle in (a); the profile of the In/Cu ratio amplifies a segregation of In at the surface. (c,d) SiNWs grown with the same 1 nm In/1 nm Cu mixed catalyst: (c) HAADF image; (d) EDX spectrum recorded in the red rectangular area in (a), showing the presence of In, Cu, Si and O in the catalyst NP; the height of the Si peak is probably due to the addition of contributions from the Cu_3Si part of the catalyst and from the nearby SiNW.

Supplementary Material, Movie S1

Effect of plasma ignition on Cu-In NPs observed *in situ* in the TEM. Catalyst NPs obtained with a nominal thickness of 0.6 nm In/0.2 nm Cu. Hydrogen is continuously introduced in the TEM column at the rate of 30 sccm, which gives a pressure 2×10^{-2} mbar; the sample temperature is 250 °C,

Supplementary Material, Movie S2

SiNW growing *in situ* in the NanoMAX TEM, with a catalyst from the In-0.6 nm/Cu-0.2 nm deposit.

1. Che, G. C.; Ellner, M., Powder crystal data for the high-temperature phases Cu4In, Cu9In4(h) and Cu2In(h). *Powder Diffr.* **2013,** *7*, 107-108. doi: 10.1017/s0885715600018340

2. Bolcavage, A.; Chen, S. W.; Kao, C. R.; Chang, Y. A.; Romig, A. D., Phase equilibria of the Cu-In system I: Experimental investigation. *J. Phase Equilib.* **1993,** *14*, 14-21. doi: 10.1007/BF02652157
